# Bidirectional Association between Nonalcoholic Fatty Liver Disease and Gallstone Disease: A Cohort Study

**DOI:** 10.3390/jcm7110458

**Published:** 2018-11-21

**Authors:** Yoosoo Chang, Yoo-Hun Noh, Byung-Seong Suh, Yejin Kim, Eunju Sung, Hyun-Suk Jung, Chan-Won Kim, Min-Jung Kwon, Kyung Eun Yun, Jin-Won Noh, Hocheol Shin, Yong Kyun Cho, Seungho Ryu

**Affiliations:** 1 Department of Occupational and Environmental Medicine, Kangbuk Samsung Hospital, Sungkyunkwan University School of Medicine, Seoul 03181, Korea; yoosoo.chang@gmail.com (Y.C.); byungseong.suh@samsung.com (B.-S.S.); 2 Center for cohort studies, Total Healthcare Center, Kangbuk Samsung Hospital, Sungkyunkwan University School of Medicine, Seoul 04514, Korea; reenya@live.co.kr (Y.K.); hs1601.jung@samsung.com (H.-S.J.); chanwon75.kim@samsung.com (C.-W.K.); ke.yun@samsung.com (K.E.Y.); hcfm.shin@samsung.com (H.S.); 3 Department of Clinical Research Design & Evaluation, SAIHST, Sungkyunkwan University, Seoul 06351, Korea; 4 Department of Anatomy and Cell Biology, College of Medicine, Chung-Ang University, Seoul 06974, Korea; 5 Department of Family Medicine, Kangbuk Samsung Hospital, Sungkyunkwan University School of Medicine, Seoul 03181, Korea; eunjusung68@gmail.com; 6 Department of Laboratory Medicine, Kangbuk Samsung Hospital, Sungkyunkwan University School of Medicine, Seoul 03131, Korea; mjk.kwon@samsung.com; 7 Department of Healthcare Management and Institute of Global Healthcare Research, Eulji University, Seongnam 13135, Korea; jinwon.noh@gmail.com; 8 Global Health Unit, Department of Health Sciences, University Medical Centre Groningen, University of Groningen, Groningen 9712, The Netherlands; 9 Division of Gastroenterology and Hepatology, Department of Internal Medicine, Kangbuk Samsung Hospital, Sungkyunkwan University School of Medicine, Seoul 03181, Korea; choyk2004.cho@samsung.com

**Keywords:** bidirectional relationship, nonalcoholic fatty liver disease, gallstones, insulin resistance, cohort study

## Abstract

Nonalcoholic fatty liver disease (NAFLD) and gallstone disease (GD) are often found to coexist but the sequential relationship of NAFLD and GD to each other remains controversial. We prospectively evaluated the bidirectional relationship of NAFLD with GD. A cohort study was performed on Korean adults who underwent a health checkup and were followed annually or biennially for a mean of 6.0 years. Fatty liver and gallstones were diagnosed by ultrasound. NAFLD was defined as hepatic steatosis on ultrasonography in the absence of excessive alcohol use or other identifiable causes. The NAFLD severity was determined by non-invasive fibrosis markers. Among 283,446 participants without either gallstones or cholecystectomy at baseline, 6440 participants developed gallstones. Among 219,641 participants without NAFLD at baseline, 49,301 participants developed NAFLD. The multivariable-adjusted hazard ratio (95% confidence interval) for incident gallstone comparing the NAFLD group vs. the non-NAFLD group was 1.26 (1.17–1.35). Increased non-invasive fibrosis markers of NAFLD were positively associated with an increased incidence of gallstones in a graded and dose-responsive manner (*p*-trend < 0.01). The multivariable-adjusted hazard ratios (95% confidence intervals) for incident NAFLD comparing gallstone and cholecystectomy to no GD were 1.14 (1.07–1.22) and 1.17 (1.03–1.33), respectively. This large-scale cohort study of young and middle-aged individuals demonstrated a bidirectional association between NAFLD and GD. NAFLD and its severity were independently associated with an increased incidence of gallstones, while GD and cholecystectomy were also associated with incident NAFLD. Our findings indicate that the conditions may affect each other, requiring further studies to elucidate the potential mechanisms underlying this association.

## 1. Introduction

Nonalcoholic fatty liver disease (NAFLD) is becoming one of the most common liver disorders in parallel with the global increase in obesity and type 2 diabetes [1]. NAFLD encompasses a spectrum of liver disorders ranging from simple steatosis to inflammatory steatohepatitis (NASH) with or without fibrosis/cirrhosis and hepatocellular carcinoma [2,3]. In addition to its liver-related complications, NAFLD is associated with significant non-liver morbidity, impaired health-related quality of life, and a higher use of health care resources [4,5]. NAFLD has been traditionally considered a consequence of metabolic syndrome (MetS), also known as insulin resistance syndrome, but the relationship of NAFLD with MetS is complex, and a growing body of evidence suggests the NAFLD-MetS relationship as being bidirectional in nature [6,7,8,9,10,11]. Gallstone disease (GD) is also a common condition whose prevalence is increasing with the ongoing rise in obesity, and represents a major epidemiologic and economic burden worldwide [12]. NAFLD and GD are commonly found to coexist [13,14,15,16] and have similar associated risk factors, including insulin resistance, obesity, metabolic syndrome, and type 2 diabetes [12,17,18]. Insulin resistance is considered a pivotal feature of both NAFLD and cholesterol gallstone [19,20]. Additionally, the severity of insulin resistance correlates with liver histology in patients with NAFLD [17,21]. Until now, to our knowledge, only two longitudinal cohort studies among Chinese and Taiwanese populations have examined the association between NAFLD and GD, without consideration of hepatic fibrosis [22,23]. Furthermore, no longitudinal cohort study has evaluated whether gallstones or cholecystectomy are associated with increased risk of developing NAFLD. Recent reports suggest that the gallbladder and bile acids appear to play a role in systemic metabolic regulation, and cholecystectomy itself may contribute to NAFLD development [18,24]. Currently, the sequential relationship of NAFLD and GD remains unclear, and, to the best of our knowledge, no longitudinal cohort data on a bidirectional association between NAFLD and GD are available. 

We examined whether NAFLD and its severity, based on noninvasive fibrosis markers, are associated with incident GD compared with no NAFLD, and sought determine whether gallstones and cholecystectomy are associated with an increased risk of developing NAFLD in a large cohort of Korean men and women who underwent a health screening program.

## 2. Methods

### 2.1. Study Population

The Kangbuk Samsung Health Study is a cohort study of South Korean adults who underwent a comprehensive annual or biennial health examination at Kangbuk Samsung Hospital Total Healthcare Center in Seoul and Suwon, South Korea [25,26]. More than 80% of the participants were employees of various companies and local governmental organizations and their spouses. In South Korea, the Industrial Safety and Health Law requires annual or biennial health screening exams for all employees, offered free of charge. The remaining participants voluntarily registered for the screening exams.

**Figure 1:**
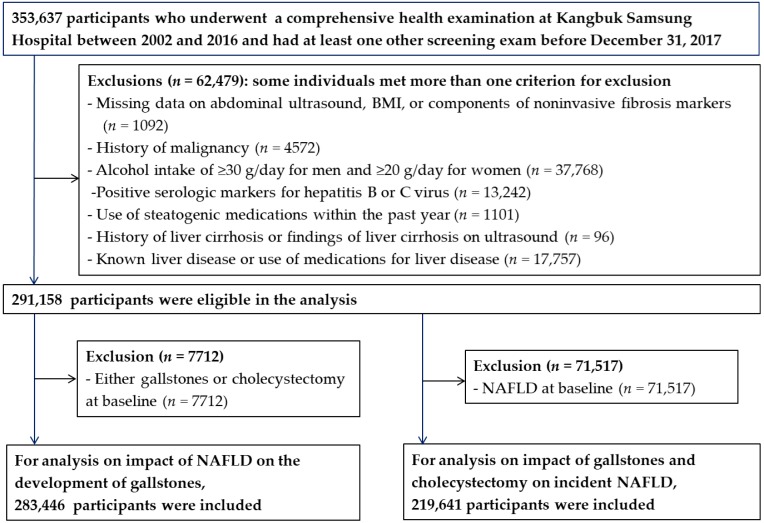
Flow diagram for the selection of study subjects.

The present analysis included study participants who underwent the comprehensive health examinations from January 1, 2002, to December 31, 2016, and who had at least one other screening exam before December 31, 2017 (*n* = 353,637; Figure 1). We excluded 62,479 subjects who had any of the following conditions at baseline: missing data for abdominal ultrasonography, body mass index (BMI), or components of noninvasive fibrosis markers (*n* = 1092); history of malignancy (*n* = 4572); known liver disease or current use of medications for liver disease (*n* = 17,757); a history of liver cirrhosis or findings of liver cirrhosis based on ultrasound (*n* = 96); alcohol intake of ≥30 g/day for men or ≥20 g/day for women [1] (*n* = 37,768); positive serologic markers for hepatitis B or C virus (*n* = 13,242); or use of medications associated with NAFLD within the past year such as valproate, amiodarone, methotrexate, tamoxifen, or corticosteroids (*n* = 1101) [1]. Because some participants met more than one exclusion criterion, a total of 291,158 participants were eligible for this study. For the analysis of the impact of NAFLD on the development of gallstones, we excluded 7712 subjects with either gallstones or cholecystectomy at baseline, leaving 283,446 to be included in the final analysis. For the analysis of the impact of gallstones and cholecystectomy on incident NAFLD, 71,517 subjects with NAFLD at baseline were excluded, leaving 219,641 to be included in the final analysis. This study was approved by the Institutional Review Board of Kangbuk Samsung Hospital, which waived the requirement for informed consent because we accessed only de-identified data routinely collected as part of health screening examinations.

### 2.2. Measurements

Baseline and follow-up examinations were conducted at the Kangbuk Samsung Hospital Total Healthcare Center [25]. Data on medical history, medication use, and health-related behaviors were collected through a self-administered questionnaire, while the physical measurements, ultrasound, and serum biochemical parameters were measured by trained staff during the health examinations. All variables were assessed at each visit.

Blood pressure, height, weight and waist circumference were measured by trained nurses. Obesity and abdominal obesity were defined as BMI ≥ 25 kg/m^2^ following Asian-specific criteria [27]. Hypertension was defined as a systolic blood pressure ≥ 140 mmHg, a diastolic blood pressure ≥ 90 mmHg, a self-reported history of hypertension, or current use of anti-hypertensive medications.

Blood specimens were sampled from the antecubital vein after the individual had undergone at least a 10-h fast. The fasting blood sample measurements included total cholesterol, low density lipoprotein-cholesterol (LDL-C), high-density lipoprotein cholesterol (HDL-C), triglycerides, alanine aminotransferase (ALT), aspartate aminotransferase (AST), gamma-glutamyltransferase (GGT), glucose, uric acid, high sensitivity C-reactive protein (hsCRP), albumin, and platelet counts.

To assess the risk of severe NAFLD, three non-invasive indices of liver fibrosis were used: NAFLD fibrosis score (NFS), fibrosis-4 (FIB-4), and aspartate transaminase to platelet ratio index (APRI). NFS was calculated according to the following published formula: NFS = −1.675 + 0.037 × age (years) + 0.094 × BMI (kg/m^2^) + 1.13 × impaired fasting glucose or diabetes (yes = 1, no = 0) + 0.99 × AST/ALT ratio − 0.013 × platelet (×10^9^/L) − 0.66 × albumin (g/dL). Two cutoff points were selected to categorize subjects with NAFLD into three groups according to their probability of advanced fibrosis: high (NFS > 0.676), intermediate (NFS: 0.676 to −1.455), and low (NFS < −1.455) [28]. The FIB-4 index was calculated by the following formula: FIB-4 = (age (years) × AST (U/L))/(platelet count (×10^9^/L) × ALT (U/L)^1/2^). For histologically defined NASH with advanced fibrosis, the area under the receiver-operating characteristic curve, sensitivity, and specificity of FIB-4 are 0.86 (95% CI, 0.78–0.94), 85%, and 65%, respectively [29]. Cut-off values from the curve were used to define low (FIB-4 < 1.30), intermediate, and high (FIB-4 ≥ 2.67) probabilities of advanced fibrosis [30]. The APRI was calculated by the following formula: APRI = 100 × (AST/upper limit of normal)/platelet count (×10^9^/L). Cut-offs for low and high probability of advanced fibrosis were 0.5 and 1.5, respectively [31]. Since a very small number of participants were identified as having NAFLD and high probability of advanced fibrosis based on non-invasive fibrosis markers, we combined the intermediate and high fibrosis score groups.

Abdominal ultrasound scans were performed by experienced radiologists, all unaware of the study aims, using a 3.5 MHz probe. Images were captured in a standard fashion with the patient in the supine position with the right arm raised above the head. The liver, gallbladder, pancreas, kidneys and spleen were evaluated in a standard fashion at each visit. An ultrasonographic diagnosis of fatty liver was determined based on known standard criteria, including a diffuse increase of fine echoes in the liver parenchyma compared with kidney or spleen parenchyma, deep beam attenuation, and bright vessel walls [32]. The inter-observer reliability and intra-observer reliability for fatty liver diagnosis were substantial (kappa statistic of 0.74) and excellent (kappa statistic of 0.94), respectively. NAFLD was defined as the presence of fatty liver in the absence of excessive alcohol use (a threshold of < 20 g/day for women and < 30 g/day for men) or other identifiable cause, as described in the exclusion criteria.

Gallstones were defined as ultrasound-documented gallstones by the presence of strong intraluminal echoes that were gravity dependent or that attenuated ultrasound transmission (acoustic shadowing) [33]. Cholecystectomy was defined as evidence of a cholecystectomy (a right upper quadrant or epigastric scar and the absence of a gallbladder) [33]. The inter-observer reliability and intra-observer reliability for gallstone diagnosis were excellent (kappa statistics of 0.90 and 0.96, respectively). 

### 2.3. Statistical Analyses

Student’s *t*-test for continuous variables with normal distribution, Kruskal-Wallis test for variables with non-normal distribution, and a Chi-square test for categorical variables were used to compare the characteristics of the study participants at baseline according to incident GD. 

Incidence density was expressed as the number of cases divided by person-years. Follow-up for each participant extended from the baseline exam until the development of the endpoint or the last health exam. Since we knew that the endpoint had developed between two visits but did not know the precise time, we used a parametric proportional hazard model to take into account this type of interval censoring (*stpm* command in Stata) [34]. In these models, the baseline hazard function was parameterized with restricted cubic splines in log time with four degrees of freedom. We estimated the adjusted hazard ratios (aHR) with 95% confidence intervals (CI) for endpoints. We assessed the proportional hazards assumption by examining graphs of estimated log(−log) survival; no violation of the assumption was found. 

#### 2.3.1. Baseline NAFLD and Incident GD

A cholecystectomy can be performed due to acalculous gallbladder diseases such as gallbladder polyps, tumors, acalculous cholecystitis, and biliary dyskinesia, which collectively represent between 5% and 30% of laparoscopic cholecystectomies [35,36]. Because the indications for the cholecystectomies were not available, we used ultrasound-documented gallstones as the primary endpoint. We estimated the adjusted hazard ratios (aHR) with 95% confidence intervals (CI) for incidental gallstones by comparing NAFLD with a low fibrosis score and NAFLD with an intermediate to high fibrosis score to the no-NAFLD group among subjects without gallstone disease at baseline. 

The models were initially adjusted for age and sex and then further adjusted for BMI, study center, year of examination, education level, smoking, alcohol intake, exercise, total calorie intake, history of hypertension, history of diabetes, and medication for dyslipidemia. To assess whether the relationship between NAFLD and incident GD was mediated by LDL-C, HDL-C, triglycerides, HOMA-IR, or hsCRP, we included those variables in multivariable models. To determine the linear trends of risk, the number of categories was used as a continuous variable and tested on each model.

We performed subgroup analysis according to the presence of obesity to evaluate the association between NAFLD and gallstones in non-obese and obese individuals, since NAFLD is closely associated with obesity, and adjustment for BMI may not be enough to control for the effects of obesity. In sensitivity analyses, we analyzed the association between NAFLD and cholecystectomy as well. We also performed a sensitivity analysis using fatty liver index as a surrogate marker of NAFLD to examine an association between smoking and incident NAFLD. The fatty liver index (FLI) was calculated according to the published formula [37]. The following cutoff values were used: FLI < 30 ruled out and FLI ≥ 60 meant fatty liver [37].

#### 2.3.2. Baseline Gallstone, Cholecystectomy and Incident NAFLD.

The primary endpoint was incident NAFLD. Since studies have reported that cholecystectomy itself was significantly associated with NAFLD, we estimated the aHR with 95% confidence intervals (CI) for incidental NAFLD separately comparing gallstone and cholecystectomy to no GD among subjects without NAFLD at baseline. The models were initially adjusted for age and sex and then further adjusted for BMI, center, year of examination, education level, smoking, alcohol intake, exercise, total calorie intake, history of hypertension, history of diabetes, and medication for dyslipidemia. To assess whether the relationship between GD and incident NAFLD was mediated by LDL-C, HDL-C, triglycerides, HOMA-IR, or hsCRP, we included those variables in the model. 

Since the association between NAFLD and gallstones can differ by sex based on previous study findings, we performed stratified analyses by sex (men vs. women). The interactions by the subgroups were tested using likelihood ratio tests comparing models with versus without multiplicative interaction terms.

Statistical analyses were performed using STATA version 15.0 (StataCorp LP, College Station, Texas, TX, USA). All *p* values were 2-tailed, and statistical significance was set at *p* < 0.05.

## 3. Results

Baseline characteristics of 283,446 participants without GD at baseline are presented according to the presence of incident GD (Table 1). At baseline, the mean (standard deviation) age and BMI of the study subjects were 37.0 (8.0) years and 23.1 (3.2) kg/m^2^, respectively; 52.4% were male, and the prevalence of NAFLD was 24.3%. NAFLD, obesity, diabetes mellitus, hypertension, BMI, glucose, blood pressure (BP), total cholesterol, triglycerides, LDL-C, hepatic enzymes, hsCRP, and HOMA-IR were positively associated with incident GD, and HDL-C and alcohol intake were negatively associated with incident GD. 

### 3.1. Baseline NAFLD and Incident GD

During 1,703,427.0 person-years of follow-up, 6640 participants developed gallstones (overall incidence rate 3.8 per 1000 person-years; 3.9 per 1000 person-years in men and 3.7 per 1000 person-years in women) (Table 2). The median follow-up period for participants was 5.0 years (interquartile range 2.4–8.9, up to 15.8 years). After adjusting for age, sex, BMI, center, year of examination, education level, smoking, alcohol intake, exercise, history of hypertension, history of diabetes, and medication for dyslipidemia, the aHR (95% CI) for incident gallstone comparing the NAFLD group vs. the no NAFLD group was 1.32 (1.22–1.43) in men and 1.35 (1.18–1.53) in women. The association persisted after adjusting for metabolic parameters including LDL-C, HDL-C, triglycerides, HOMA-IR, or hsCRP. The risk for incidental gallstone did not vary significantly by gender (*p* for interaction = 0.745). In subgroup analysis stratified by the presence of obesity, defined as BMI ≥ 25 kg/m^2^, NAFLD was significantly associated with increased risk of incident gallstone in both non-obese and obese individuals (Appendix A).

Table 3 shows the association between NAFLD and its severity based on non-invasive fibrosis markers and the development of gallstones. In multivariate-adjusted models, an increase across baseline NAFLD categories based on NFS predicted an increase in the incidence of gallstones in a graded and dose-responsive manner (*p*-rend < 0.01). The aHRs (95% CI) for gallstones comparing NAFLD with low NFS and NAFLD with intermediate or high NFS vs. no NAFLD were 1.30 (1.21–1.39) and 1.54 (1.30–1.82), respectively (Table 3 and model 1). Using other fibrosis markers based on FIB-4 and APRI, the results were similar for NAFLD with a low fibrosis score and NAFLD with an intermediate or high score. In the sensitivity analysis (Appendix B), the association of NAFLD based on fatty liver index with incident gallstone was similarly observed.

We also examined the association of NAFLD with the risk for incidental cholecystectomy or a combined endpoint including either gallstone or cholecystectomy (Appendix C). The association between NAFLD and the development of either gallstone or cholecystectomy was observed in both men and women. However, the association between NAFLD and incident cholecystectomy tended to be stronger in women than in men (*p* for interaction = 0.033).

### 3.2. Baseline Gallstone, Cholecystectomy and Incident NAFLD

During 1,165,454.7 person-years of follow-up, 49,301 participants developed NAFLD (overall incidence rate 42.3 per 1000 person-years; 68.0 per 1000 person-years in men and 23.5 per 1000 person-years in women) (Table 4). For men, multivariable-adjusted HR (95% CI) for incident NAFLD comparing GD and cholecystectomy to no GD were 1.12 (1.03–1.22) and 1.21 (1.04–1.42), respectively, while for women. the corresponding HR (95% CI) were 1.23 (1.13–1.35) and 0.98 (0.80–1.19), respectively. The increased risk for incidental NAFLD with cholecystectomy was evident in men but not in women (*p* for interaction = 0.002).

## 4. Discussion

In this large-scale cohort study of young and middle-ged individuals, we examined the bidirectional relationship between NAFLD and GD during a median of 5 years of follow-up. We found that in one direction, NAFLD was associated with an increased risk of developing GD, and in the other direction, gallstone and cholecystectomy were associated with an increased risk of incident NAFLD. Using the non-invasive fibrosis markers, subjects with NAFLD and intermediate or high NFS had the highest incidence of gallstones, but even with a low probability of hepatic fibrosis based on fibrosis markers, NAFLD was significantly associated with the development of gallstones. These associations persisted even after adjusting for possible confounders, lipid profiles, HOMA-IR, and hsCRP, suggesting bidirectional and independent relationships exist between NAFLD and gallstones.

Previous studies, which have included mostly cross-sectional studies and only a few cohort studies, have evaluated the association between NAFLD and GD, but their relationship remains controversial [38,39,40]. While some of these cross-sectional studies showed an increased prevalence of GD in patients with NAFLD, others reported a relationship in the other direction, showing an increased prevalence of NAFLD in patients with GD or cholecystectomy [13,14,15,16,38,41,42]. Until now, only two cohort studies on the association between NAFLD and GD were available [22,23]. A cohort study of 11,200 Chinese health checkup examinees over 6 years demonstrated that NAFLD was associated with an increased incidence of gallstones, with a stronger association in female participants [23]. The other cohort study, of 1296 Chinese adults with a mean follow-up of 3.51 years, also reported a positive association of NAFLD with GD (22). Likewise, both studies reported that NAFLD predicts the development of GD. However, in a recent pilot study of non-obese, middle-aged patients, liver fat as shown by abdominal magnetic resonance imaging was significantly increased in patients who had undergone cholecystectomy 2 years ago (*n* = 26) compared to normal patients (*n* = 16) [43]. Although the association between NAFLD and GD has been hypothesized to be bidirectional, no cohort studies have examined this hypothesis before our study. To the best of our knowledge, this is by far the largest longitudinal cohort study demonstrating a prospective bidirectional relationship between NAFLD and GD, while accounting for a considerable number of possible confounders. NAFLD was independently associated with an increased risk for developing gallstones in both men and women. Even in nonobese men and women, positive association between NAFLD and incident gallstones was observed; thus, the presence of obesity and other metabolic factors could not fully explain these associations. Indeed, NAFLD, even in nonobese individuals, is associated with insulin resistance, impaired glucose tolerance, and metabolic syndrome, all of which are risk factors for GD [11,44,45,46]. Additionally, NAFLD overproduces cholesterol and alters cholesterol metabolism independent of obesity, which might contribute to the formation of cholesterol gallstones [11,47,48].

With regard to NAFLD severity, a recent cross-sectional study in patients with biopsy-proven NAFLD reported that the prevalence of GD increased with advancing fibrosis [41]. In our study, increasing severity of NAFLD from low to intermediate or high NFS at baseline was associated with higher incidence of gallstones in a dose-responsive manner, but even NAFLD with low NFS was also associated with a higher incidence of gallstones when compared with no NAFLD. However, the small number of patients with a high probability of fibrosis in the study did not allow a separate category for a more severe form of NAFLD.

In the other direction, we also demonstrated that both GD and cholecystectomy were associated with an increased risk of developing NAFLD. A significantly increased risk of incident NAFLD was observed in both men and women with gallstones but only in men with cholecystectomy. Even though the number of female participants was large, the incidence of NAFLD was much lower in women than in men (23.5 per 1000 person-years in women and 68.0 per 1000 person-years in men), resulting in a lack of power to detect an association between cholecystectomy and incident NAFLD in the relatively lean and young female participants.

Though there have been only limited data on the incidence rate of GD among the general population, the incidence of gallstones was lower in our study (3.9 per 1000 person-years in men and 3.7 per 1000 person-years in women) than in other populations [49,50]. This difference could be explained by age and ethnic differences. The frequency of gallstones increases with age, escalating markedly after 40 years of age by 4–10-fold [50], and in our study, 72.9% of the subjects were younger than 40 years. Ethnically, a lower prevalence of GD has been reported for Asian populations compared to Western populations [51]. Regarding gallstone composition, pigment stones still comprise a relatively higher proportion of gallstones in East Asians; however, the epidemiological and composition characteristics of GD have become similar to those seen in Western countries [52]. If pigment stones are included in this study, the resultant association between NAFLD and gallstones may be diluted due to the different pathogenesis of gallstone types. 

The mechanisms underlying the bidirectional association between NAFLD and gallstones are incompletely understood. Insulin resistance, a key feature of NAFLD development and progression, could play a major role in the pathogenesis of gallstones by favoring the production of cholesterol-supersaturated bile, inducing a lithogenic bile salt profile and altering gallbladder function, all of which are key features in the pathogenesis of cholesterol gallstones [19,38]. Indeed, the production of bile supersaturated with cholesterol from the liver is a key early metabolic event underlying cholesterol lithogenesis [53]. NAFLD is characterized by disordered lipid metabolism, inhibition of fatty acid oxidation, and enhanced lipogenesis [53]. Therefore, these metabolic milieus in NAFLD may trigger pathophysiologic processes associated with gallstone formation. However, the relationship between insulin resistance and GD may not be unilateral, since gallbladder dysfunction has been associated with NAFLD and other insulin resistance-associated conditions [38,43]. In our study, even after adjustment for HOMA-IR, the bidirectional association between NAFLD and gallstones persisted. Several systemic metabolic changes following cholecystectomy have been linked to the pathophysiology of NAFLD in previous studies [38]. Glucose and lipid metabolism may be affected by alterations in bile acid metabolism in the absence of gall bladder, contributing to development of NAFLD [54]. The altered circulation of bile acids exerts effects on hepatic lipid and glucose metabolism modulated via activity of bile acid receptors such as the farnesoid X receptor and TGR5, leading to gene expression changes in the liver that may lead to development of NAFLD [55,56]. Another possible mechanism is the decreased level of fibrosis growth factor 19 (FGF19), which is mainly secreted by gall bladder mucosa, after cholecystectomy [57]. FGF19 regulates the *de novo* synthesis of bile salts, lipogenesis, and energy homeostasis [58,59] and has been shown to have inhibitory effects on hepatic fatty acid synthesis [60,61]. Therefore, decreased FGF19 level following cholecystectomy may alter metabolic regulation, favoring triglyceride accumulation in the liver [58,62]. In fact, lower serum level of FGF19 was found to be associated with increased risk of NAFLD [63]. Further prospective studies are warranted to elucidate the mechanisms for an increased risk of NAFLD after cholecystectomy.

In our study, the association between NAFLD and development of gallstone was similarly observed in both men and women, whereas the association between NAFLD and incident cholecystectomy tended to be stronger in women than in men (*p* for interaction = 0.033). NAFLD, a sexually dimorphic disease, more often affects men, but gallstones are more common in women [64,65]. Similarly, in our study, men were found to be more likely to have NAFLD, while women were more likely to develop gallstones. Previous studies showed that the prevalence of cholecystectomy was generally higher in women, which was also consistent across different ethnic groups [33,66]. The pronounced risk of incident cholecystectomy in women with NAFLD seen in our study might be correlated to a higher likelihood of having symptomatic GD in women, but its mechanism is not clearly understood. Previous studies have reported the stronger association of obesity with the risk of symptomatic GD in women than in men, possibly due to the role of estrogen secreted by adipose tissue [67,68]; estrogen has also been linked to the increased risk of gallstones and cholecystectomy in women [69,70]. Similarly, NAFLD and the effect of estrogen in women might act additively or synergistically in contributing to the development of symptomatic gallstones. However, further studies are needed to fully explain the role of NAFLD in the development of gallstones as well as the role of gallstones or cholecystectomy in the development of NAFLD while considering the existence of different effects by sex.

The study had several limitations. First, NAFLD was diagnosed based on ultrasound results, while liver biopsy is regarded as the gold standard. However, ultrasound is highly accurate for steatosis and is widely used clinically and in population-based studies [71]. Second, the information on the indications for cholecystectomy was not available; thus, we could not differentiate cholecystectomy unrelated to gallstones [35,36]. However, the majority of gallstones are not associated with symptoms [72]; thus, incidental asymptomatic gallstones as a separate outcome in our study could lead to a better understanding of the development of gallstones. Finally, our findings from relatively healthy young and middle-aged Korean men and women may not be generalizable to other populations with different ages or race/ethnicity, or in different settings.

The major strength of our study was that NAFLD and gallstones diagnosed with ultrasound were assessed repeatedly over time along with other confounders, which allowed us to evaluate the temporal association between NAFLD and the development of asymptomatic gallstones. In addition to the large sample size, our study population was relatively young and healthy, and thus our findings may be less likely to be affected by confounding or selection biases due to comorbidities. 

## 5. Conclusions

In conclusion, this cohort study demonstrated a bidirectional and longitudinal relationship between NAFLD and GD. NAFLD and non-invasive fibrosis markers were independently associated with an increased incidence of gallstones, while gallstones and cholecystectomy were also associated with incident NAFLD. Our findings indicate that the conditions may affect each other, requiring further studies to elucidate the potential mechanisms underlying this association.

## Figures and Tables

**Table 1:** Baseline characteristics of study participants according to incident gallstones by sex (*n* = 283,446).

Characteristic	Men (*n* = 148,593)		*p* Value	Women (*n* = 134,853)		*p* Value
	No Incident Gallstones	Incident Gallstones		No Incident Gallstones	Incident Gallstones	
Number	144,948	3645		132,058	2795	
Age (years) ^a^	37.0 (7.8)	37.9 (8.3)	<0.001	36.9 (8.3)	36.8 (7.7)	0.260
Current smoker (%)	38.0	40.4	0.004	2.2	2.5	0.303
Alcohol intake (%) ^b^	45.1	40.5	<0.001	7.4	6.0	0.007
Vigorous exercise (%) ^c^	15.0	12.8	<0.001	12.7	11.8	0.141
Higher education (%) ^d^	86.8	85.9	0.210	72.5	71.1	0.141
Fatty liver (%)	37.2	48.6	<0.001	9.7	17.5	<0.001
Diabetes mellitus (%)	3.0	4.0	<0.001	1.4	2.0	0.008
Hypertension (%)	15.2	19.0	<0.001	5.6	7.5	<0.001
Medication for dyslipidemia (%)	1.2	1.3	0.490	0.9	0.7	0.303
Obesity (%)	37.4	48.1	<0.001	12.1	23.5	<0.001
BMI (kg/m^2^)	24.3 (2.9)	25.1 (3.0)	<0.001	21.7 (2.9)	22.8 (3.6)	<0.001
Systolic BP (mmHg) ^a^	115.9 (12.2)	117.3 (12.4)	<0.001	105.3 (12.8)	107.8 (14.0)	<0.001
Diastolic BP (mmHg) ^a^	74.9 (9.3)	76.4 (9.4)	<0.001	67.1 (9.0)	68.7 (9.7)	<0.001
Glucose (mg/dL) ^a^	95.2 (14.7)	96.4 (16.9)	<0.001	90.9 (11.5)	92.4 (13.7)	<0.001
Total cholesterol (mg/ dL) ^a^	197.8 (34.2)	201.7 (36.0)	<0.001	185.8 (32.8)	187.3 (34.2)	0.022
LDL-C (mg/ dL) ^a^	121.5 (30.2)	123.0 (30.8)	0.002	106.1 (28.5)	108.0 (29.5)	<0.001
HDL-C (mg/ dL) ^a^	52.2 (11.5)	49.9 (10.7)	<0.001	63.0 (14.1)	59.4 (13.4)	<0.001
Triglycerides (mg/ dL) ^e^	116 (82–166)	129 (93–186)	<0.001	73 (56–100)	82 (60–116)	<0.001
Albumin (g/dL) ^a^	4.7 (0.2)	4.6 (0.2)	<0.001	4.5 (0.2)	4.5 (0.2)	0.342
AST (U/L) ^e^	23 (19–28)	24 (20–29)	<0.001	18 (16–22)	19 (16–22)	<0.001
ALT (U/L) ^e^	24 (18–35)	28 (20–41)	<0.001	14 (11–18)	15 (12–20)	<0.001
GGT (U/L) ^e^	26 (18–41)	31 (20–48)	<0.001	12 (9–16)	13 (10–19)	<0.001
hsCRP (mg/L) ^e^	0.5 (0.3–1.0)	0.7 (0.4–1.3)	<0.001	0.3 (0.1–0.7)	0.4 (0.2–1.0)	<0.001
HOMA-IR ^e^	1.6 (1.1–2.2)	1.9 (1.3–2.5)	<0.001	1.4 (0.9–2.0)	1.7 (1.2–2.3)	<0.001

Data are ^a^ mean (standard deviation); ^e^ median (interquartile range), or percentage; ^b^ ≥ 10 g of ethanol per day; ^c^ ≥ 3 times per week; ^d^ ≥ college graduate; Abbreviations: ALT, alanine aminotransferase; AST, aspartate aminotransferase; BMI, body mass index; BP, blood pressure; GGT, gamma-glutamyltransferase; HDL-C, high-density lipoprotein-cholesterol; hsCRP, high sensitivity C-reactive protein; HOMA-IR, homeostasis model assessment of insulin resistance; LDL-C, low-density lipoprotein cholesterol.

**Table 2:** The associations between nonalcoholic fatty liver disease (NAFLD) and the development of gallstones.

	Number	Person-Years	Incident Case	Incidence Density (1000 Person-Year)	Age- and Sex-Adjusted HR ^a^ (95% CI)	Multivariate HR ^a^ (95% CI)	
						Model 1	Model 2
Total (*n* = 283,446)							
No NAFLD	214,446	1,295,745.6	4180	3.2	1.00 (reference)	1.00 (reference)	1.00 (reference)
NAFLD	69,000	407,681.4	2260	5.5	1.77 (1.68–1.87)	1.31 (1.22–1.40)	1.26 (1.17–1.35)
Men (*n* = 148,593)							
No NAFLD	121,518	605,332.7	2307	3.1	1.00 (reference)	1.00 (reference)	1.00 (reference)
NAFLD	13,335	339,409.0	488	5.2	1.67 (1.57–1.79)	1.32 (1.22–1.43)	1.26 (1.15–1.37)
Women (*n* = 134,853)							
No. NAFLD	92,928	690,412.9	1873	3.3	1.00 (reference)	1.00 (reference)	1.00 (reference)
NAFLD	55,665	68,272.5	1772	7.1	2.17 (1.96–2.41)	1.35 (1.18–1.53)	1.28 (1.11–1.46)

^a^ Estimated from parametric proportional hazard models. The *p* value for the interaction of sex and NAFLD on the risk of incident gallstones was 0.740. Multivariable adjusted model 1 was adjusted for age, sex, BMI, center, year of examination, education level, smoking, alcohol intake, exercise, total calorie intake, history of hypertension, history of diabetes and medication for dyslipidemia, except sex in the stratified analysis by sex; model 2: model 1 plus adjusted for LDL-C, HDL-C, triglycerides, HOMA-IR, or hsCRP. Abbreviations: BMI, body mass index; CI, confidence intervals; HDL-C, high-density lipoprotein-cholesterol; HR, hazard ratios; hsCRP, high sensitivity C-reactive protein; HOMA-IR, homeostasis model assessment of insulin resistance; LDL-C, low-density lipoprotein cholesterol; NAFLD, nonalcoholic fatty liver disease.

**Table 3:** The associations between nonalcoholic fatty liver disease (NAFLD) and its severity based on non-invasive fibrosis markers and the development of gallstones.

	Number	Person-Years	Incident Case	Incidence Density (1000 Person-Year)	Age- and sex-Adjusted HR ^a^ (95% CI)	Multivariate HR ^a^ (95% CI)	
						Model 1	Model 2
Based on NFS							
No NAFLD	214,446	1,295,745.6	4180	3.2	1.00 (reference)	1.00 (reference)	1.00 (reference)
NAFLD, Low NFS	63,985	384,074.4	2056	5.4	1.73 (1.64–1.83)	1.30 (1.21–1.39)	1.25 (1.16–1.34)
NAFLD, Intermediate or high NFS	5015	23,607.0	204	8.6	2.40 (2.07–2.78)	1.54 (1.30–1.82)	1.50 (1.26–1.78)
*p* for trend					<0.001	<0.001	<0.001
Based on FIB 4							
No NAFLD	214,446	1,295,745.6	4180	3.2	1.00 (reference)	1.00 (reference)	1.00 (reference)
NAFLD, Low FIB 4	65,526	392,234.6	2157	5.5	1.77 (1.68–1.88)	1.31 (1.23–1.41)	1.26 (1.17–1.36)
NAFLD, Intermediate or high FIB 4	3474	15,446.8	103	6.7	1.68 (1.37–2.06)	1.20 (0.95–1.51)	1.21 (0.96–1.53)
*p* for trend					<0.001	<0.001	<0.001
Based on APRI							
No NAFLD	214,446	1,295,745.6	4180	3.2	1.00 (reference)	1.00 (reference)	1.00 (reference)
NAFLD, Low APRI	64,537	381,044.8	2101	5.5	1.76 (1.67–1.86)	1.31 (1.22–1.40)	1.26 (1.17–1.36)
NAFLD, Intermediate or high APRI	4463	26,636.6	159	6.0	1.89 (1.61–2.22)	1.31 (1.10–1.57)	1.26 (1.05–1.50)
*p* for trend					<0.001	<0.001	<0.001

**^a^** Estimated from parametric proportional hazard models. Multivariable adjusted model 1 was adjusted for age, sex, BMI, center, year of examination, education level, smoking, alcohol intake, exercise, total calorie intake, history of hypertension, history of diabetes and medication for dyslipidemia; model 2: model 1 plus adjusted for LDL-C, HDL-C, triglycerides, HOMA-IR, or hsCRP. Abbreviations: APRI, aspartate transaminase to platelet ratio index; CI, confidence intervals; FIB-4, fibrosis 4; HDL-C, high-density lipoprotein-cholesterol; HR, hazard ratios; hsCRP, high sensitivity C-reactive protein; HOMA-IR, homeostasis model assessment of insulin resistance; LDL-C, low-density lipoprotein cholesterol; NAFLD, nonalcoholic fatty liver disease; NFS, NAFLD fibrosis score.

**Table 4:** The associations between gallstone, cholecystectomy and the development of nonalcoholic fatty liver disease (NAFLD) (*n* = 219,641).

	Number	Person-Years	Incident Case	Incidence Density (1000 Person-Year)	Age- And Sex-Adjusted HR ^a^ (95% CI)	Multivariate HR ^a^ (95% CI)	
						Model 1	Model 2
Total (*n* = 219,641)							
No gallstone disease	214,446	1,141,715.8	47,992	42.0	1.00 (reference)	1.00 (reference)	1.00 (reference)
Gallstone	4073	19,015.1	1051	55.3	1.36 (1.28–1.44)	1.16 (1.09–1.24)	1.14 (1.07–1.22)
Cholecystectomy	1122	4723.8	258	54.6	1.23 (1.09–1.39)	1.10 (0.97–1.25)	1.17 (1.03–1.33)
Men (*n* = 94,865)							
No gallstone disease	92,928	484,621.6	32,782	67.6	1.00 (reference)	1.00 (reference)	1.00 (reference)
Gallstone	1494	6653.3	570	85.7	1.26 (1.16–1.37)	1.12 (1.03–1.22)	1.10 (1.01–1.20)
Cholecystectomy	443	1763.4	154	87.3	1.29 (1.10–1.51)	1.21 (1.04–1.42)	1.29 (1.10–1.52)
Women (*n* = 124,776)							
No gallstone disease	121,518	657,094.2	15,210	23.1	1.00 (reference)	1.00 (reference)	1.00 (reference)
Gallstone	2579	12,361.8	481	38.9	1.53 (1.40–1.68)	1.23 (1.13–1.35)	1.15 (1.05–1.27)
Cholecystectomy	679	2960.5	104	35.1	1.17 (0.96–1.42)	0.98 (0.80–1.19)	1.05 (0.86–1.28)

^a^ Estimated from parametric proportional hazard models. The *p*-value for the interaction of sex and gallstone disease on the risk of incident NAFLD was 0.002. Multivariable adjusted model 1 was adjusted for age, sex, BMI, center, year of examination, education level, smoking, alcohol intake, exercise, total calorie intake, history of hypertension, history of diabetes, and medication for dyslipidemia, except sex in the stratified analysis by sex; model 2: model 1 plus adjustment for LDL-C, HDL-C, triglycerides, HOMA-IR, or hsCRP. Abbreviations: CI, confidence intervals; HDL-C, high-density lipoprotein-cholesterol; HR, hazard ratios; hsCRP, high sensitivity C-reactive protein; HOMA-IR, homeostasis model assessment of insulin resistance; LDL-C, low-density lipoprotein cholesterol; NAFLD, nonalcoholic fatty liver disease.

## Appendix A

**Table A1:** The associations between nonalcoholic fatty liver disease (NAFLD) and development of gallstones by the presence of obesity, defined as BMI ≥ 25 kg/m^2^.

	Number	Person-Years	Incident Case	Incidence Density (1000 Person-Years)	Age- and Sex-Adjusted HR ^a^ (95% CI)	Multivariate HR ^a^ (95% CI)	
						Model 1	Model 2
Men							
BMI < 25 kg/m^2^							
No NAFLD	72,546	473,899.9	1333	2.8	1.00 (reference)	1.00 (reference)	1.00 (reference)
NAFLD	20,146	122,294.6	560	4.6	1.59 (1.44–1.75)	1.53 (1.37–1.71)	1.38 (1.22–1.56)
BMI ≥ 25 kg/m^2^							
No NAFLD	20,382	131,432.8	540	4.1	1.00 (reference)	1.00 (reference)	1.00 (reference)
NAFLD	35,519	217,114.3	1212	5.6	1.37 (1.24–1.52)	1.37 (1.22–1.53)	1.24 (1.10–1.40)
Women							
BMI < 25 kg/m^2^							
No NAFLD	112,024	635,857.6	1969	3.1	1.00 (reference)	1.00 (reference)	1.00 (reference)
NAFLD	6230	32,165.6	168	5.2	1.70 (1.45–1.99)	1.64 (1.37–1.97)	1.36 (1.12–1.65)
BMI ≥ 25 kg/m^2^							
No NAFLD	9494	54,555.3	338	6.2	1.00 (reference)	1.00 (reference)	1.00 (reference)
NAFLD	7105	36,106.9	320	8.9	1.51 (1.30–1.76)	1.42 (1.19–1.69)	1.38 (1.14–1.66)

^a^ Estimated from parametric proportional hazard models. The *p* value for the interaction of obesity and NAFLD for the risk of incident gallstones was 0.300 in women, and 0.170 in men. Multivariable adjusted model 1 was adjusted for age, sex, center, year of examination, education level, smoking, alcohol intake, exercise, total calorie intake, history of hypertension, history of diabetes, and medication for dyslipidemia; model 2 included model 1 plus adjustments for LDL-C, HDL-C, triglycerides, HOMA-IR, and hsCRP. Abbreviations: BMI, body mass index; CI, confidence interval; HDL-C, high-density lipoprotein cholesterol; HR, hazard ratio; hsCRP, high-sensitivity C-reactive protein; HOMA-IR, Homeostasis Model Assessment of Insulin Resistance; LDL-C, low-density lipoprotein cholesterol; NAFLD, nonalcoholic fatty liver disease.

## Appendix B

**Table A2:** The associations between fatty liver index (FLI) and the development of gallstones (*n* = 194,666).

	Number	Person-Years	Incident Case	Incidence Density (1000 Person-Year)	Age- and Sex-Adjusted HR ^a^ (95% CI)	Multivariate HR ^a^ (95% CI)	
						Model 1	Model 2
Total							
FLI <30	142,201	686,999.9	2197	3.2	1.00 (reference)	1.00 (reference)	1.00 (reference)
FLI 30–<60	34,267	173,595.0	876	5.0	1.70 (1.55–1.85)	1.31 (1.17–1.46)	1.22 (1.08–1.38)
FLI ≥60	18,198	89,353.3	572	6.4	2.25 (2.03–2.49)	1.47 (1.27–1.71)	1.29 (1.08–1.54)
*p* for trend					<0.001	<0.001	0.002
Men							
FLI <30	52,982	272,555.5	795	2.9	1.00 (reference)	1.00 (reference)	1.00 (reference)
FLI 30–<60	29,360	152,751.1	712	4.7	1.51 (1.36–1.67)	1.29 (1.13–1.48)	1.21 (1.05–1.41)
FLI ≥60	16,556	82,794.1	491	5.9	1.98 (1.77–2.21)	1.52 (1.27–1.82)	1.30 (1.04–1.63)
*p* for trend					<0.001	<0.001	0.013
Women							
FLI <30	89,219	414,444.4	1402	3.3	1.00 (reference)	1.00 (reference)	1.00 (reference)
FLI 30–<60	4907	20,843.9	164	7.8	2.36 (2.00–2.79)	1.38 (1.11–1.71)	1.24 (0.98–1.56)
FLI ≥60	1642	6559.2	81	12.3	3.83 (3.05–4.81)	1.46 (1.05–2.04)	1.31 (0.91–1.89)
*p* for trend					<0.001	0.004	0.074

^a^ estimated from parametric proportional hazard model. The *p*-value for the interaction of sex and FLI on the risk of incident gallstone was 0.503. Multivariable adjusted model 1 was adjusted for age, sex, BMI, center, year of examination, education level, smoking, alcohol intake, exercise, total calorie intake, history of hypertension, history of diabetes and medication for dyslipidemia, except sex in the stratified analysis by sex; model 2: model 1 plus adjusted for LDL-C, HDL-C, triglycerides, HOMA-IR, or hsCRP. Abbreviations: CI, confidence intervals; FLI, fatty liver index; HDL-C, high-density lipoprotein-cholesterol; HR, hazard ratios; hsCRP, high sensitivity C-reactive protein; HOMA-IR, homeostasis model assessment of insulin resistance; LDL-C, low-density lipoprotein cholesterol; NAFLD, nonalcoholic fatty liver disease.

## Appendix C

**Table A3:** The associations between nonalcoholic fatty liver disease (NAFLD) and the development of gallstones or cholecystectomy (*n* = 283,446).

	Number	Person-Years	Incident Case	Incidence Density (1000 Person-Year)	Age- and Sex-Adjusted HR ^a^ (95% CI)	Multivariate HR ^a^ (95% CI)	
						Model 1	Model 2
Gallstone or cholecystecomty							
Total							
No NAFLD	214,446	1,299,654.6	4927	3.8	1.00 (reference)	1.00 (reference)	1.00 (reference)
NAFLD	69,000	409,552.0	2612	6.4	1.73 (1.64–1.82)	1.28 (1.20–1.37)	1.24 (1.16–1.33)
Men							
No NAFLD	92,928	607,142.6	2207	3.6	1.00 (reference)	1.00 (reference)	1.00 (reference)
NAFLD	55,665	340,888.9	2036	6.0	1.63 (1.53–1.73)	1.29 (1.20–1.39)	1.23 (1.14–1.34)
Women							
No NAFLD	121,518	692,512.0	2720	3.9	1.00 (reference)	1.00 (reference)	1.00 (reference)
NAFLD	13,335	68,663.1	576	8.4	2.11 (1.93–2.32)	1.33 (1.18–1.50)	1.28 (1.13–1.45)
Cholecystectomy							
Total							
No NAFLD	214,446	1,311,709.6	1150	0.9	1.00 (reference)	1.00 (reference)	1.00 (reference)
NAFLD	69,000	415,994.8	556	1.3	1.56 (1.40–1.74)	1.10 (0.96–1.26)	1.10 (0.95–1.27)
Men							
No NAFLD	92,928	613,204.5	543	0.9	1.00 (reference)	1.00 (reference)	1.00 (reference)
NAFLD	55,665	346,147.1	424	1.2	1.40 (1.24–1.59)	1.06 (0.90–1.25)	1.04 (0.88–1.24)
Women							
No NAFLD	121,518	698,505.0	607	0.9	1.00 (reference)	1.00 (reference)	1.00 (reference)
NAFLD	13,335	69,847.7	132	1.9	2.03 (1.67–2.47)	1.30 (1.01–1.68)	1.26 (0.96–1.64)

^a^ Estimated from parametric proportional hazard model. Multivariable adjusted model 1 was adjusted for age, sex, BMI, center, year of examination, education level, smoking, alcohol intake, exercise, total calorie intake, history of hypertension, history of diabetes, and medication for dyslipidemia, except sex in the stratified analysis by sex; model 2: model 1 plus adjusted for LDL-C, HDL-C, triglycerides, HOMA-IR, or hsCRP. Abbreviations: CI, confidence intervals; HDL-C, high-density lipoprotein-cholesterol; HR, hazard ratios; hsCRP, high sensitivity C-reactive protein; HOMA-IR, homeostasis model assessment of insulin resistance; LDL-C, low-density lipoprotein cholesterol; NAFLD, nonalcoholic fatty liver disease. The *p*-value for the interaction of sex and NAFLD on the risk of incident gallstone disease (gallstone or cholecystectomy) was 0.511. The *p*-value for the interaction of sex and NAFLD on the risk of incident cholecystectomy was 0.033.

## Appendix D

**Table A4:** The factors associated with NAFLD and GD at baseline before prospective longitudinal analysis from which persons with NAFLD or GD at baseline were excluded.

	Multivariable-Adjusted Odds Ratios (95% CI) for NAFLD	Multivariable-Adjusted Odds Ratios (95% CI) for GD
Age per 10-year increment	1.16 (1.14–1.19)	1.71 (1.65–1.76)
Male	2.49 (2.40–2.59)	0.61 (0.56–0.66)
Suwon center	1.38 (1.34–1.41)	1.04 (0.98–1.11)
year of screening exam per 1-year	1.06 (1.05–1.06)	1.04 (1.03–1.05)
Education level ≥ college graduate	1.15 (1.11–1.19)	1.31 (1.22–1.42)
Alcohol intake		
<10 g of ethanol per day	0.88 (0.85–0.91)	0.95 (0.89–1.02)
≥10 g of ethanol per day	0.72 (0.69–0.75)	0.86 (0.79–0.94)
Smoking		
Ever smoker	1.01 (0.97–1.05)	1.08 (0.99–1.19)
Never smoker	0.93 (0.89–0.96)	1.13 (1.04–1.24)
Vigorous exercise ≥ 3 times per week	0.82 (0.79–0.85)	0.99 (0.91–1.07)
History of hypertension	1.10 (1.05–1.16)	0.96 (0.86–1.06)
History of diabetes	1.82 (1.65–2.02)	1.01 (0.85–1.20)
Medication for dyslipidemia	1.62 (1.44–1.83)	0.89 (0.72–1.10)
BMI per 1 SD increment	2.74 (2.69–2.79)	1.23 (1.19–1.27)
LDL-C per 1 SD increment	1.33 (1.32–1.35)	0.96 (0.93–0.99)
HDL-C per 1 SD increment	0.73 (0.72–0.74)	0.93 (0.90–0.96)
TC per 1 SD increment	1.59 (1.57–1.62)	0.93 (0.89–0.96)
HOMA-IR per 1 SD increment	1.71 (1.68–1.74)	1.15 (1.11–1.18)
hsCRP per 1 SD increment	1.30 (1.28–1.31)	1.14 (1.11–1.18)

Estimated from logistic regression models. Multivariate models are adjusted for all other variables listed for the model.
